# Bowel Affections of Young Children

**Published:** 1875-12

**Authors:** S. B. Hawley

**Affiliations:** Aurora, Ill.


### EXAMINER.

Vol. XXXII. — DECEMBER, 1875. — No. 12.

### Original Communications.

### BOWEL AFFECTIONS OF YOUNG CHILDREN.

By S. B. HAWLEY, M.D., Aurora, Ill.

I am prompted by the article on this subject by Dr. N. S. Davis, in the October number of the Journal and Examiner, to write the following queries and opinions of my own, in the hope that they may quicken inquiry and discovery in a much needed direction, namely, as to how to prevent the great mortality of infants.

Dr. Davis seems to proceed upon the hypothesis that the fearful death rate of infants in the hot weeks of summer is owing to an inherent weakness or delicacy of the human organization during the infantile period, whereby it cannot successfully withstand the heat of summer. The idea that heat was the direct cause of cholera infantum, and that keeping infants cool was the true preventive, was also advocated at the State Society at Peoria, some five years since, by the late Dr. Young, of this city. Dr. Davis has well shown the coincidence of high temperature and great mortality, and we can readily believe that heat is the remote cause. But if it be the direct cause, why do we not observe a similar tendency in the other branches of the animal kingdom at the same time? Why should not high temperatures acting on their tender organizations produce like results? Again, why cannot human judgment do as well for the young of the race as instinct does for the brute? And if it cannot, why should we not study the condition of the brute closely and see if we may not thereby discover a method of approximating their healthy condition?

We observe that the brute has a uniform diet for its young, or nearly so, and that it wears a *fixed and very considerable thickness of clothing* through all the hottest weather. The brute creation seem to be more fixed to certain latitudes than man. They seem to have less power of adaptation. Man, as a race, has greater longevity. Then why should his young be swept away as with the besom of destruction? All will doubtless agree that it is because his faulty methods of protection are not equal to that which nature does for the brute—but where is the fault? I have from my own observation and reflection concluded that a *very great and telling fault was the want of sufficient protection to the skin of the infant in hot weather*. And for several years past I have been of the opinion that all bowel complaints, in children possessed of anything like a good constitution, would be easily and readily manageable if *sufficient clothing were worn at all times* to surely prevent any suppression of the natural function of the skin. I reasoned that on a hot day the infant skin is called into extra activity, and that it is so excessively sensitive to the first changes that the children in effect "take cold" before their seniors are aware of the fall of temperature, and often in the night when all are asleep there is increased likelihood of the same result. Besides, there may be, and doubtless are, some poisonous emanations vaporized by the heat, which, allowed to come in contact freely with the exposed sensitive and active skin of the infant, are absorbed, and thus contribute a share to the fearful results that we witness with each recurring summer. Acting upon these opinions, I have insisted with all mothers and nurses that I could influence, that children should sleep on the hottest nights in some thick flannel or cotton-flannel night-dress, with legs and arms so made that they cannot be thrown off, and during the hot days wear a suit of flannel in addition to the usual amount of muslin clothing commonly deemed sufficient. There is often considerable objection on account of supposed discomfort to the child, but I ask who can point out any considerable harm that the human family has suffered from over clothing at any time. I think we shall be compelled to conclude ultimately that the weakness and degeneracy of our American race is more owing to insufficient clothing than to any other one cause. But in children, so far as I have been able to learn upon careful inquiry during the five years last past, there has not occurred a single case of cholera infantum or seriously obstinate and troublesome diarrhœa or dysentery, in families that have followed my course in regard to the clothing of infants and children. I cannot regard it as an accidental occurrence, for cases, and fatal cases, of all these diseases have been numerous in our city each summer.

Whether the keeping of the skin at all times covered does most, by preventing "taking cold" or by preventing the absorption by the skin of poisonous matters, in warding off the summer diseases of children, may be a question. But on this one point I am fully convinced that keeping the bodies and limbs of infants thickly covered in the hottest weather, is an efficient means of preventing a large amount of sickness which they suffer under the prevalent management. If the clothing be of a porous kind, as flannel, evaporation through it will afford all the needful cooling for health; indeed I cannot help repeating the thought that we have very slight evidence of any considerable loss of health by over clothing in any age of human life. Dr. Davis' recommendation of removal during the hot weather to the lake or hill tops, is only available for the few. The additional clothing which I advocate is within the reach of the poorest.

I cannot now fortify my statements of opinion with definite statistics, but I can safely say my observation extends to hundreds of our city families.

